# The Tri-State Medical Society

**Published:** 1903-11

**Authors:** 


### EDITORIAL NOTES AND COMMENTS.

The Business office of THE JOURNAL-RECORD is No. 1007-1008 Century Building.

The Editorial office is 318-319-320 The Prudential.

Address all Business communications to Dr. M. B. Hutchins, Mgr.

Make remittances payable to THE ATLANTA JOURNAL-RECORD OF MEDICINE.

On matters pertaining to the Editorial and Original Communications address Dr. Bernard Wolff, Atlanta.

Reprints of original articles will be furnished at cost price. Requests for the same should always be made *on the manuscript*.

We will present, post-paid, on request, to each contributor of an original article, twenty (20) marked copies of THE JOURNAL-RECORD containing such article.

### THE TRI-STATE MEDICAL SOCIETY.

THE fifteenth annual meeting of the Tri-State Medical Society of Georgia, Alabama and Tennessee took place in this city on Tuesday, Wednesday and Thursday, October 13, 14, 15. The meetings were held in the Kimball House ball-room, and there were about one hundred members in attendance.

The following is the program. Many of the papers, owing to the absence of the writers, were read by title:

TUESDAY, OCTOBER 13—MORNING SESSION.

Registrations, Introductions, etc., 9:00 to 10:00.

Prayer by Rev. C. B. Wilmer.

Welcome address, by Hon. Evan P. Howell, Mayor of Atlanta.

Reading and discussion of papers.

Marion McH. Hull, Atlanta, Ga., "Colic in Infants."

George Brown, Atlanta, Ga., "Tuberculosis."

J. B. Murfree, Murfreesboro, Tenn., "Treatment of Cancers."

Y. L. Abernathy, Chattanooga, Tenn., "Tuberculosis."

Louis Edelman, Huntsville, Ala., "Tuberculosis and Syphilis Among the Jews."

M. M. Saliba, Savannah, Ga., "The Prevention of Pulmonary Consumption."

J. M. Johnson, Chattanooga, Tenn., "The Medical Examiner, and His Relation to the Life Insurance Company."

J. W. Macquillan, Chattanooga, Tenn., "Scleroderma"—Presentation of patient.

German Haymore, Chattanooga, "Acute Coryza."

R. H. Tatum, Chattanooga, "Physiologic Enzymes."

J. A. Simpson, Glenn Mary, Tenn., "Cerebro-Spinal Meningitis: Report of a Case."

G. Manning Ellis, Chattanooga, "Landry's Paralysis."

C. S. Durand, Chattanooga, "Sequelæ of Diphtheria."

Seale Harris, Union Springs, Ala., "Prognosis in Valvular Disease of the Heart."

Hazel Padget, Columbia, Tenn., "A General Review of Nephritis."

Claud A. Smith, Atlanta, Ga., "Nephritis."

George E. Pettey, Memphis, Tenn., "Strychnia in the Treatment of Morphinism and in other Diseases when Occurring in Morphine Habitues."

AFTERNOON SESSION—2:30-5.

Reading and discussion of papers.

J. W. Hooper, Roanoke, Ala., "Chronic Pancreatitis with Autopsy." "A Family Epidemic of Uncinariasis."

H. L. Appleton, Center, Ala., "Is Crime a Disease? With Some Suggestions for Its Cure."

E. B. Block, Atlanta, Ga., "Remarks on Dactylitis Cyphilitica."

W. D. Travis, Covington, Ga., "Variola."

L. W. Johnson, Tuskegee, Ala., "Some Cardiac Diseases with Report of Cases."

S. Kirkpatrick, Selma, Ala., "Some Unrecognized Symptoms of Eye Strain."

T. A. Casey, Birmingham, Ala., "Should the Public be Educated in Matters Medical."

M. Goltman, Memphis, Tenn., "Renal Decortication as a Cure for Chronic Nephritis."

W. G. Harrison, Talladega, Ala., "Some Recent Work in Uncinariasis."

Raymond Wallace, Chattanooga, Tenn., "Chronic Gastritis: A Comparatively Infrequent Disease."

Claude C. Pierce, Tampa Bay Quarantine, Fla., U. S. M. H. S., "The U. S. Public Health and Marine Hospital Service."

WEDNESDAY, OCTOBER 14—MORNING SESSION—9-1.

Reading and discussion of papers.

E. B. Ward, Selma, Ala., "An Unusual Case of Appendicitis."

H. A. Milford, Culpepper, Va., "The Surgical Treatment of Appendicitis."

C. Holtzclaw, Chattanooga, Tenn., "Appendicitis."

E. H. Bradford, Boston, Mass., "The Treatment of Congenital Dislocation of the Hip, with and without Incision."

Joel E. Goldthwait, Boston, "The Differential Diagnosis and Treatment of Rheumatoid Diseases."

M. C. McGannon, Nashville, "Misleading Symptoms in Abdominal Surgery," Illustrated by Case Reports.

T. M. McIntosh, Thomasville, Ga., "Report of Fourteen Cases of Lateral Lithotomy."

W. H. Wilder, Birmingham, Ala., "Treatment of Compound Fracture."

S. W. Pryor, Chester, S. C., "A Weak Solution of Cocaine or a Local Anesthetic for Most Cases of Minor Surgery."

Frank Cordle, Trion Factory, Ga., "Castration for Tuberculosis or the Testicle."

James M. Black, Knoxville, Tenn., "Cysts of the Pancreas with Report of Cases."

D. S. Middleton, Rising Fawn, Ga., "Fracture of the Spine: Report of a Case."

Willis F. Westmoreland, Atlanta, Ga., "Ulcers."

Ira J. Sellers, Birmingham, Ala., "Affections of the Gall Bladder."

AFTERNOON SESSION—2:30-5.

Reading and discussion of papers.

President's Address, "The Surgical Treatment of Lateral Curvature of the Spine."

Harvey Cushing, Baltimore, "Remarks on Recent Advances in Cerebral Localization, with Especial Remarks to the Operative Treatment of Focal Epilepsy."

William T. Berry, Birmingham, "The Treatment of Hernia in the Young."

Mark H. O'Daniel, Bullards, Ga., "Circumcision as a Means of Hygiene and Preventive of Reflex Phenomena."

G. A. Baxter, Chattanooga, "Modification of Plaster Splint for Fracture of the Thigh."

Geo. R. West, Chattanooga, "Surgical Shock."

R. N. Taylor, Chattanooga, "Inguinal Adenitis."

W. A. Duncan, Chattanooga, "Phlebitis: Report of a Case."

A. W. Stirling, Atlanta, Ga., "Some Interesting Affections of the Ocular Nerves."

Dunbar Roy, Atlanta, Ga., "The Cause and Treatment of Watery Eyes in Infants."

J. M. Crawford, Atlanta, "Empyema of the Antrum—Complications."

J. H. Shorter, Macon, Ga., "What Should be Done with a Blind Eye."

M. M. Stapler, Macon, Ga., "Stapler's Baritier and why it Should be Adopted in the Treatment of the Ear."

THURSDAY MORNING, OCTOBER 15—9-1:

T. H. Bilbro, Murfreesboro, Tenn., "The Management of the Puerperal Woman."

C. V. Stephenson, Carrollton, Tenn., "The Proper Course to Pursue on Diagnosing Pregnancy in the Unmarried."

Cornelius McCane, Savannah, Ga., "Carcimona Uteri; its Probable Cause, Treatment and Results."

R. R. Kime, Atlanta, "The Use of Natural Processes in Correcting Retro-Deviations of the Uterus."

S. W. Purifoy, Birmingham, Ala., "Retroversion of the Uterus—Is it a Symptom or a Disease."

John P. Stewart, Attalla, Ala., "Wonderful Results from the Use of the Curette."

W. L. Nolan, Chattanooga, "Ventral Fixation, Its Limitations and Results."

Edward B. Anderson, Chattanooga, "Modern Surgery."

W. G. Bogart, Chattanooga, "Obstetrical Difficulties."

Monroe Smith, Atlanta, Ga., "Treatment of Empyema."

On Tuesday evening the Society visited the horse show which was given in connection with the State Fair now in progress, as guests of the Atlanta Society of Medicine, and on Wednesday a reception was tendered it by Dr. J. M. Crawford, at his home on Peachtree Road.

The proposition to extend the scope of the Society was postponed for further and more definite action of the next meeting in Chattanooga. The officers elected for the ensuing year were as follows: President, Dr. Sloan, of Alabama; Vice-President, J.

M. Crawford, Atlanta; Dr. Frank Trester Smith was re-elected Secretary.

Among the notable visitors were Drs. E. H. Bradford and Joel E. Goldthwait, of Boston, among the most prominent orthopedic surgeons of this country. Dr. Bradford presented a paper on the treatment of congenital dislocation of the hip, with and without incision. This is a subject of especial interest owing to the *réclame* given it by the visit to this country of Prof. Lorenz, the recollection of which is fresh in the mind of every one.

